# A Mixed-Methods Approach Using Self-Report, Observational Time Series Data, and Content Analysis for Process Analysis of a Media Reception Phenomenon

**DOI:** 10.3389/fpsyg.2019.01666

**Published:** 2019-07-24

**Authors:** Michael Brill, Frank Schwab

**Affiliations:** Department of Media Psychology, Faculty of Human Sciences, Institute Human-Computer-Media, Julius-Maximilians-Universität Würzburg, Würzburg, Germany

**Keywords:** presence, measurement, blinking, structure, mixed methods

## Abstract

Due to the complexityof research objects, theoretical concepts, and stimuli in media research, researchers in psychology and communications presumably need sophisticated measures beyond self-report scales to answer research questions on media use processes. The present study evaluates stimulus-dependent structure in spontaneous eye-blink behavior as an objective, corroborative measure for the media use phenomenon of spatial presence. To this end, a mixed methods approach is used in an experimental setting to collect, combine, analyze, and interpret data from standardized participant self-report, observation of participant behavior, and content analysis of the media stimulus. T-pattern detection is used to analyze stimulus-dependent blinking behavior, and this structural data is then contrasted with self-report data. The combined results show that behavioral indicators yield the predicted results, while self-report data shows unpredicted results that are not predicted by the underlying theory. The use of a mixed methods approach offered insights that support further theory development and theory testing beyond a traditional, mono-method experimental approach.

## Introduction

### Background

Experimental research in media psychology and communications focuses on complex human experiencing and behavior, and often uses complex theorizing to explain respective phenomena. To examine these theories, researchers regularly use complex stimuli, such as movies, video games, or virtual reality environments. In addition to this inherent complexity, further challenges can arise if researchers are not only interested in outcomes of media use, but also in the dynamic process aspects of media use phenomena. In this case, summative, post-session self-report scales may not be sufficient to describe the dynamic nature of media use phenomena. Instead, more sophisticated measurement instruments, such as continuously recorded observational measures can be used to corroborate self-report measures.

In this article, we describe a study on the media use phenomenon of spatial presence, or “the conviction of being located in a mediated environment” (Wirth et al., 2007, p. 495), and put particular focus on the study’s mixed methods aspects. The goal of the study is to evaluate structural aspects of spontaneous eye-blink behavior as a possible objective, corroborative measure for presence. Based on theoretical assumptions on the emergence of presence from attentional and other cognitive processes on the one hand, and research findings on the sensitivity of spontaneous eye-blink timing to attentional and other cognitive processes on the other hand, we test if presence experiences co-occur with a higher degree of stimulus-dependent blink timing during reception of a movie as an externally valid media stimulus.

### Mixed Methods Approach

To address this research question, it is necessary to consider subjective viewer experience, objectively observable viewer behavior, and aspects of the media stimulus. The present study collects, integrates, and analyzes data from all three involved domains: First, self-report data on presence experiences is collected with an established, standardized questionnaire instrument (Spatial presence experience scale, SPES; Hartmann et al., 2015), that also serves as a validation standard for the blink-related measure. Second, the media users’ spontaneous eye-blink behavior is recorded with observational methods. Third, data on the media stimulus is obtained from two different approaches to content analysis with a focus on content-blind, computational quantification of change in visual information, and on theory-derived, presence-relevant cinematic techniques. Data from observation and content analysis is then jointly analyzed with the method of T-pattern detection (Magnusson, 1996, 2000; Casarrubea et al., 2015, 2018) to determine the degree of stimulus-dependent structure in blinking behavior, which is then compared to the self-report measure.

Regarding the mixed methods aspect, the approach in the present study is in accordance with existing guidelines and research approaches. The study is in line with guidelines for the conduction of observational studies for behavior analysis in the movement sciences (Anguera et al., 2012, 2017; Castañer et al., 2013), and also with media-related applications of mixed methods research, such as studies on the analysis of children’s movement quality during use of exergames (Castañer et al., 2011, 2016). In the present study, participant behavior in the context of media use is, as a first step, captured in video recordings. Second, computer-assisted coding using an *ad hoc* observational instrument produces descriptions of blinking behavior as annotated, sequential, qualitative time-series data. This set of data then allows data quality control and quantitative analysis with T-pattern detection to obtain structured categorical data which are, finally, further analyzed with quantitative methods.

Consequently, the mixed methods approach in the present study is characterized by collecting, analyzing, and interpreting quantitative and qualitative data to obtain deeper insights into a phenomenon of interest (Leech and Onwuegbuzie, 2009). By providing a precise description of the media stimulus, and by describing and analyzing the relation between self-reported recipient experience and observed recipient behavior during media use, the study aims at obtaining a more holistic understanding of the phenomenon under research (Venkatesh et al., 2013). Moreover, the sequential analysis procedure with quantitative and qualitative data allows for an unconventional analysis approach (Anguera et al., 2018). In this case, the approach aims at the analysis of temporal structure in user behavior and its relation to self-reported experience, thus providing a process-oriented perspective on the presence phenomenon.

### Spatial Presence

Presence can be defined as the perceptual illusion of non-mediation (Lombard and Ditton, 1997). Wirth et al. (2007) model the emergence of spatial presence as part of a fluctuating, binary state, in which media users either enter a state of presence and temporarily overlook the mediated nature of their media use experience or they do not. In a state of presence, media users experience themselves as located within the medium and their action possibilities as determined by the medium. Further, the user’s mental capacities are assumed to be no longer bound by the real environment in which media use takes place, but by the mediated environment. Wirth et al. (2007) propose a two-stage process model for the formation of spatial presence: First, the user needs to construct a spatial situation model (SSM) from spatial cues presented by the medium. This first step is supported by media factors that attract automatic attention allocation, by user factors that support controlled attention allocation on the medium, and by additional media and user factors that support the creation of an SSM. Once an SSM has been established, the new SSM rivals the user’s existing SSM of the real environment. According to the model, presence occurs after a second step, if processes under influence of further user factors and media factors result in the viewer favoring the mediated SSM over the real-world SSM as the viewer’s primary ego-reference-frame.

Wirth et al. (2007) point out that presence formation is a continuous process, since it depends on repeated confirmation of the medium as the primary ego-reference-frame. It is necessary for this process that the medium presents a stable stream of coherent information that the user continuously attends to and processes this information, and that the SSM is continuously updated. In this process, the model sees attention as a necessary, but not sufficient condition for the emergence of presence: automatic and controlled attention support the intake of media information, so that the further cognitive processes are provided with the necessary information. Continuous attention allocation occurs with both “short-term orienting responses and more persistent attention allocation” (Wirth et al., 2007, p. 499), and media factors can support attention allocation, for example, by presenting topics that match the user’s domain-specific interest in media content, or by rapid stimulus changes in media form. Attention is also a crucial component of the model after creation of an SSM, when the mediated SSM is repeatedly tested against the real-world SSM. Then, attention and other cognitive processes related to SSM updating and testing of the rivaling situation models are engaged in continued processing of mediated information.

A sensible way to assess subjective presence experiences is self-report (Wirth et al., 2008), which is commonly achieved by means of standardized self-report scales (e.g., Usoh et al., 2000; Lessiter et al., 2001; Hartmann et al., 2015). However, inherent limitations of questionnaires have led researchers to criticize the self-report approach (e.g., Usoh et al., 2000; Slater, 2004; Slater and Garau, 2007; Liebold et al., 2017), in particular when focusing on the process nature of the presence phenomenon (Brill, 2019). To overcome these limitations, several objective indicators for presence experiences have been tested. For example, Böcking et al. (2008) report evaluations of think aloud, eye-tracking, secondary task reaction time, and functional magnetic resonance imaging. Other researchers focused on breaks in presence, that is, instances at which the users become aware of the mediated nature of their experience, and have used a range of psychophysiological measures to objectively describe observable correlates of user experience (Slater et al., 2003; Rey et al., 2011). Liebold et al. (2017) linked experimentally induced orienting responses on formal media features to breaks in presence, and observed concomitant changes in psychophysiology and blink timing. In the present study, we examine structural aspects of spontaneous eye-blink behavior as an indicator for presence-related processing of a stimulus.

### Spontaneous Eye-Blink Behavior

Spontaneous eye-blinks are a ubiquitous behavior that serves the physiological function of maintaining clarity of vision and tear film stability (Cruz et al., 2011). However, research suggests that blinks are not purely following physiological necessities, but are subject to other internal and external influences, as well. Researchers have described blinking behavior as an indicator for cognitive activity (Malmstrom et al., 1977), and have found that blink timing depends on complexity and attentional demands of a task (Stern et al., 1984). Blinking has been described as an indicator for, or possibly even a component of processes around shifts in cognitive state (Oh et al., 2012). Blinks have been seen as an indicator for cognitive processing, possibly for finalized stimulus evaluation in tasks with and without need for motor responses, and as a “reliable marker of cognitive processing speed even in no-go situations” (Wascher et al., 2015, p. 1216). Blinks have even been attributed an active role in cognitive processing: they may possibly support switching between internal and external orienting networks, and could thus facilitate memory retrieval and support information processing by contributing to attention disengagement (Nakano, 2015).

Specifically for media use, Nakano et al. (2009) observed synchronized blinking behavior in recipients of video stories as a natural film stimulus. Synchronization effects were found as an intra-individual effect, whereas recipients showed similar blinking behavior during repeated viewing of the stimulus, and as an inter-individual effect, whereas recipients of the same stimulus independently showed similar blink timing. Nakano et al. (2009) assumed that the mechanism for blink synchronization would primarily depend on content aspects of the presented story, such as implicit break-points in the story, and not so much on low-level stimulus aspects, such as cuts. Synchronization effects were not found for a video stimulus without explicit story, or for an audio stimulus with story. Nakano et al. (2009) thus assumed the existence of “a mechanism for controlling the timing of blinks that searches for the appropriate timing to prevent the loss of critical information from the flow of visual information” (p. 3642). Nomura et al. (2015) related structural aspects of blinking behavior during reception of comedy performances to self-reported transportation experience, and also found synchronization effects in the blinking behavior of recipients, including differences between novice and advanced users of the stimulus.

### Study Rationale

Based on the body of research on presence, on the adaptive nature of blink timing, and on media-use-related synchronization phenomena of spontaneous eye-blink behavior, we hypothesize a link between presence-related processes and blinking behavior. If recipients continuously allocate their attention on the media stimulus and engage in continued cognitive processing of media content, then the impact of the respective processes on blink timing should lead to a higher degree of stimulus-dependent structure in spontaneous eye-blink behavior. If, on the other hand, users do not engage in attention on and cognitive processing of a media stimulus, with their attentional and cognitive resources operating independently from the stimulus content, then their blink timing should be more independent from the media stimulus.

Regarding the question which exact features of the stimulus contribute to the structuring of blinking behavior, the present study tests two possible stimulus features. First, optical flow (Burton and Radford, 1978) as a low-level, content-blind feature of stimulus form is tested. With high amounts of optical flow, that is, a high change rate in visual information, a large amount of new visual information is presented to the recipient, and this information can be used to update or reconstruct the recipient’s SSM. The cognitive activity during intake and processing of this information should affect blink timing. Second, cinematic form is tested as a factor of influence. Media content is rarely presented as unedited, raw footage from an observing camera perspective, but rather makes use of a cinematic, formal language that supports presentation of narrative content (Ohler, 1994; Zacks et al., 2010; Magliano and Zacks, 2011; Monaco, 2015). Parallel to the consideration of optical flow, the second approach to content analysis also aims at identifying instances in the stimulus where a reconstruction or updating of the recipient’s SSM would be necessary. However, the second approach uses the definition of the *scene* in films (Monaco and Bock, 2011, p. 239) to identify relevant events in cinematic structure. As existing media research shows, T-pattern detection can be used to analyze movie-viewers’ anticipations of, and reactions toward a film’s moments of impact and narrative structure (Suckfüll and Unz, 2013, 2016). Further, the latter research informs the present study since it used T-pattern detection to relate media events to observe psychophysiological and behavioral variables in order to infer on internal processes in viewers, in this case raising and disappointing of viewer expectations.

## Materials and Methods

Following the rationale of existing evaluation studies for objective presence indicators (Böcking et al., 2008), we conducted an experiment with a between-subjects design, in which participants were randomly assigned to conditions with either a high, or a low presence potential. Because established experimental manipulations to increase a medium’s presence potential, such as presentation in stereoscopic 3D or larger screen size (see Cummings and Bailenson, 2016, for a meta-analysis of technological presence factors), or to decrease presence potential, such as dual task paradigms (e.g., Böcking et al., 2008), could possibly affect blinking behavior, a visually neutral manipulation with identical stimulus was intended. The intended manipulation considered the distinction between media form and media content (e.g., Ohler, 1994), and the assumption that attention to aspects of media form will interfere with presence formation (Liebold et al., 2017): Media recipients should not experience presence if they pay attention to the medium itself, because then, they should be unable to overlook the mediated nature of their experience. Therefore, a manipulation by different instructions intended to hinder or support presence formation by directing the participants’ attention to the narrated media content, or to media form features. While participants in the high-presence condition were asked to consume the stimulus as they would regularly do in a cinema, participants in the low-presence condition were explicitly asked to pay attention to and analyze cinematic form features of the stimulus. The manipulation effectiveness was controlled with short, custom items that were designed to ask for the participants’ attentional focus and reception mode (Vorderer, 1992; Suckfüll, 2004) during the reception situation, and with a standardized presence questionnaire (Spatial presence experience scale, SPES; Hartmann et al., 2015).

This approach represents a rigorous testing of the hypotheses, because both experimental groups presented an identical stimulus, and observed differences in behavioral structure of blinking can be attributed to different attention allocation on, and processing of this stimulus. In addition, this approach is superior to use a non-media low presence condition, since spatial presence in the sense of the theory (Wirth et al., 2007) can only emerge during media use.

### Stimulus

As a natural media stimulus, we presented the first 20:23 min from the academy award-winning motion picture *Birdman or (The Unexpected Virtue of Ignorance)* (Iñárritu, 2014). The movie is produced without visible cuts or transitions, so the camera follows the actors through sets in fluid movements, and no abrupt changes by cuts disrupt the flow of visual information. As in Nakano et al.’s (2009) experiment, a baseline video was presented prior to the actual stimulus. Here, we used a 1:55 min long video showing a large fish tank in a public aquarium (Rawlinson, 2009). Before, in between, and after the two videos, a 5 s countdown on the screen served as a transition indicator.

### Apparatus

The experiment was conducted in a cinema-like, acoustically optimized, darkened laboratory with identical technical setup for all sessions. The stimulus video was available in Blu-ray quality, and was presented on a 150″ projection screen at a viewing distance of 4.3 m in upscaled 4 K resolution. Audio was presented with a room-calibrated 7.2 channels surround sound system. Synchronized observations of the screen and of participants were recorded with unobtrusively placed near-infrared pan-tilt-zoom dome cameras and the Noldus Media Recorder 2 software. Before each session, temperature and relative humidity were recorded with a consumer-grade digital thermometer and hygrometer. Environmental conditions in the laboratory were relatively stable (temperature: *M* = 20.26°C, SD = 0.27, range: 19.50 – 20.90; relative humidity: *M* = 39.79%, SD = 1.82, range: 38.20 – 44.00).

### Participants

The study was carried out in accordance with the research institution’s ethical guidelines. All participants provided written informed consent in accordance with the Declaration of Helsinki. Sixty-one participants (age *M* = 22.39 years, SD = 2.73, range: 19 – 33 years, 63.9% female) with normal or corrected to normal vision participated in the study, and were granted course credit, if desired. Twenty participants or 32.8% of the sample were already familiar with the stimulus; however, due to existing findings on intra-subject eye-blink synchronization during repeated viewing of a stimulus (Nakano et al., 2009; Nomura et al., 2015), these participants were still included in the study. Exclusion criteria were a very high blink rate of more than 60 blinks per second or more than two standard deviations above the sample’s mean, reporting no sufficient vision acuity, failure to comply with experimental instructions and procedures, or not declaring or retracting consent to participate. Depending on the recruitment situation, group-size of the different experimental sessions varied between 1 and 5 participants (group size in high presence condition: *n* = 13; *M* = 2.31, SD = 1.32; low presence condition: *n* = 16; *M* = 1.94, SD = 1.12).

### Measures

To collect self-report data, post-session questionnaires were handed out in print in two versions with different, randomized item sequence within scales. The Spatial presence experience scale with its sub-scale “spatial presence: self-location” (SPES; Hartmann et al., 2015) with 5-point Likert scales served as the standardized measure for subjective presence self-reports. Four additional short items were used as manipulation check, and asked for their focus on content aspects, focus on media form aspects, and for the degree of involved and reflective reception mode (Vorderer, 1992).

**Figure 1:**
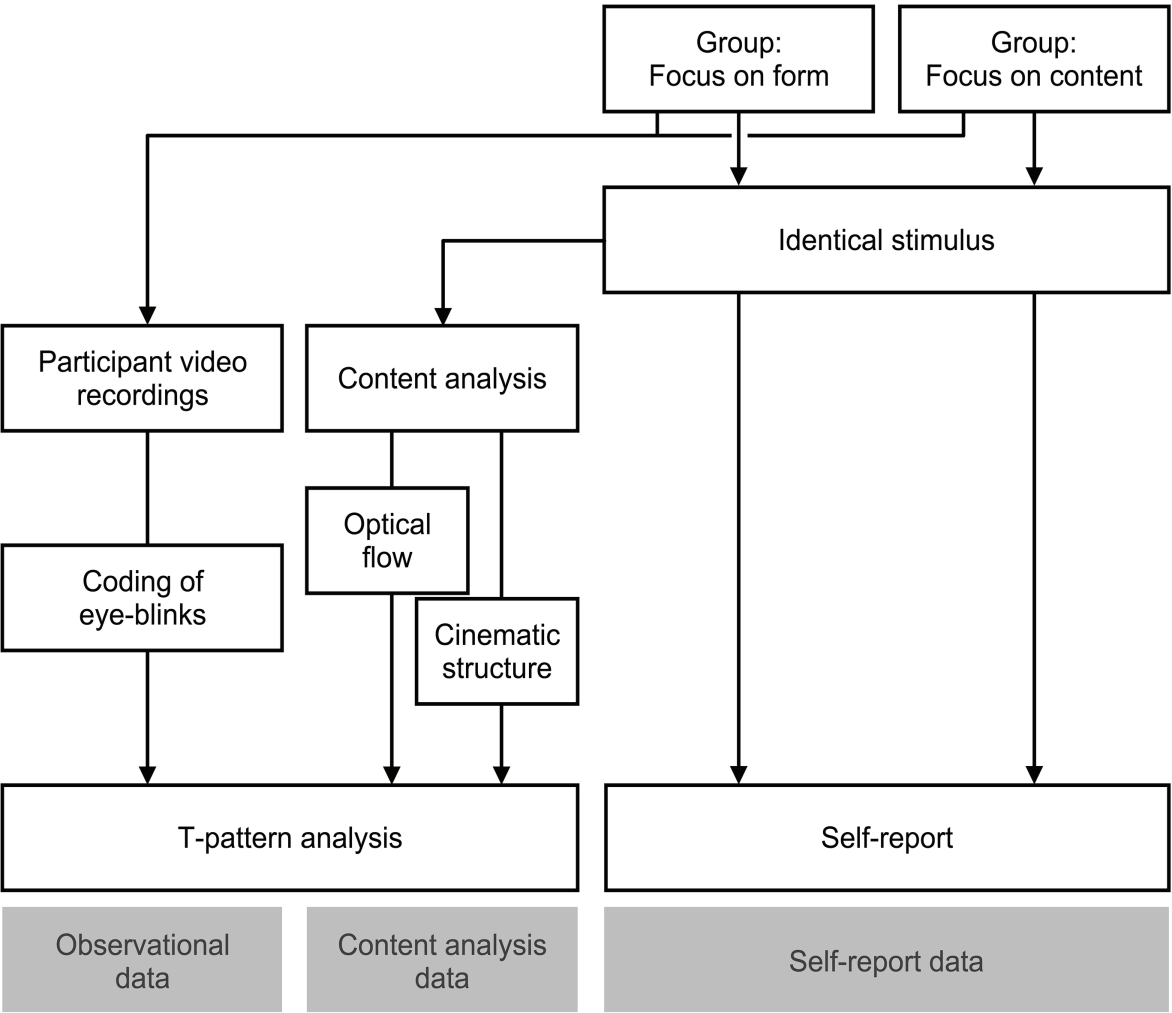
Schematic overview of the experimental and analytical procedures in the present study’s mixed methods approach.

To collect observational data, videos of the individual participants were recorded in non-participative observation during presentation of baseline video and stimulus video. The observer had only standardized contact with participants during the instruction phase, and was not present in the room during the observation period. Participant recordings were captured in HD resolution with 30 frames per second, and were either recorded from a slight downward angle or in half profile, so blinks were clearly visible (see Figure 1 for an overview of experimental and analytical procedures).

### Procedure

After participants had been introduced to the experimental setting and procedures and declared informed consent, the experimenter instructed them with a standardized text, and then started video recordings and stimulus presentation from a separate room. After the video presentation, recordings were stopped, the participants answered the questionnaire, and were then debriefed and dismissed (see Figure 1).

### Coding and Data Preparation

#### User Behavior

Several participants were excluded from behavior coding and subsequent analysis. One participant reported abnormal blinking behavior due to a foreign object in the eye; four participants did not comply with instructions on conduct during the experiment; two participants were excluded from coding, because it became obvious in an early stage of coding that their blink rate throughout the session was near or above 60 blinks per minute. This reduced the sample size for behavior coding to 53 participants.

Blinks were coded manually by two independent coders with the Noldus Observer XT 11.5 software. Blinks were to be coded if a rapid downward movement of the upper eyelids, followed by re-opening of the lids, covered at least part of the pupils. The video frame with maximum eyelid closure was coded as the time of blink occurrence. Coder one coded the whole observation periods of each participant with baseline video and stimulus video. Coder two coded a randomly determined subsample of the same recordings. To this end, a random timestamp was generated for each participant recording, and from that timestamp on, 10% of the participant’s observation period were coded by the second coder.

Concordance between the two independent coders for the 10% subsample of the observation period was calculated for a 170 ms or five frames tolerance window. Mean percentage of agreement was 92.72% (SD = 8.64, range: 64.30 − 100). Average inter-rater agreement with Cohen’s kappa was very low (*M* = 0.15, SD = 0.39, range: −0.2 − 1). Because very low kappa has been reported to possibly occur under certain conditions despite high agreement percentage (Feinstein and Cicchetti, 1990), the codings were nevertheless accepted.

The 53 participants showed during baseline, transitions and stimulus video a total of 24,361 blinks. During stimulus presentation, the participants showed 22,546 blinks, with an average blink rate of 20.88 blinks per minute (SD = 11.07). Four participants were excluded from further analyses because their blink rate was more than two standard deviations above the coded sample’s mean.

#### Media Events: Optical Flow

For the first type of media events, optical flow in the stimulus video was analyzed to identify instances with a high change rate in visual information. To quantify optical flow, the Birdman excerpt was processed with the *OpenCV* toolbox (Bradski, 2000; OpenCV, 2014) and an included algorithm for the detection of optical flow (Farnebäck, 2003). The algorithm was used to compute optical flow between all consecutive frames of the stimulus video. The resulting vector with optical flow values between video frames was processed further in *GNU Octave* (Eaton and Octave Community, 2015; Eaton et al., 2015a,b): first, the signal was smoothed with a 12 frames or 0.5 s moving average filter. Then, the *findpeaks* function from the Octave signal processing package (Various authors and Miller, 2015) was used to identify peaks with a height of at least two standard deviations of the detrended, smoothed optical flow vector, with a minimum peak width of one frame, and a minimum distance between peaks of 48 frames or 2 s. According to visual inspection, these parameters represented the signal’s peaks sufficiently well. The detection algorithm identified 55 peaks. These peaks were numbered consecutively, and their timestamps were integrated with individual blink time stamps from behavior coding.

This computational approach can be used to analyze video material efficiently, and is useful to test assumptions according to which blink timing is sensitive to the flow of information in the visual channel. However, this approach is content blind, since it is irrelevant what exactly caused optical flow in the stimulus. Therefore, in order to gain more interpretative capacity, we also coded theoretically derived media events in the stimulus video to increase content sensitivity of analyses.

#### Media Events: Cinematic Structure

The second approach to content analysis focused on formal aspects of cinematic structure to identify instances in the stimulus that could cause reconstruction or updating of the SSM. Starting from the definition of the *scene* as a basic unit of film that consists of one or more shots connected by place, plot, or present persons (Monaco and Bock, 2011, p. 239), we derived several events that present new, SSM-relevant information to the viewer: First, changes of location, that is, a new room is shown in the video. Second, the camera movements of camera panning, tracking shots and spinning shots, because they make visible new parts of the mediated environment. Third, the emergence of new characters interacts with the protagonist. In addition, unpredicted events in the movie’s story were included, because they present new information which needs to be attended to, and needs to be integrated in the situation model. A code book was created with categories, definitions, coding rules, and coding examples. Two coders assessed the Birdman sequence independently in the Noldus Observer XT 11.5 software, and coded onsets for all relevant media events. Due to the fluid changes of most aspects, event onsets were less defined, so a larger tolerance window of 2 s was used for calculation of coder agreement. Initial codings showed a low coder agreement of 59.1%, with a Cohen’s kappa of *κ* = 0.49. Independent recoding of disagreements led to a coder agreement of 90.1%, with a Cohen’s kappa of *κ* = 0.87 with a 2 s tolerance window. For the final definition of media events, all cases of agreement were used, resulting in 183 events: 40 changes of location, 64 panning shots, 5 spinning shots, 51 tracking shots, 18 appearances of characters, and 5 unexpected events. With respect to the subsequent T-pattern detection, the earlier timestamp of both codings was used to define event onsets, because different timing in a sequence of events would have less impact on analysis than different order in a sequence. As for peaks in optical flow, the events in each category were numbered in sequence, and all media events were integrated with individual eye-blink data for T-pattern analysis.

### T-Pattern Analysis

To test the hypothesis that users in the presence-supporting content condition would show a higher degree of stimulus-dependent structure in spontaneous eye-blink behavior, T-pattern analysis (Magnusson, 1996, 2000) was used to analyze temporal relationships between user events and media events. T-pattern analysis is based on repeated, binomial-statistics-based analysis steps that identify higher-than-chance co-occurrences of events. This process identifies a T-Pattern, if an event B follows an event A with greater than chance timing within a critical interval. In a level-by-level process, identified T-patterns are used as new events in subsequent analysis steps, so the analysis allows for the identification of complex hierarchical structures. Going beyond other methods of group statistics or sequential analysis, the T-Pattern method enables researchers to analyze, for example, structural aspects of human behavior in dyadic interaction (Magnusson, 2000), in the interaction within and between sports teams (Lozano et al., 2016; Fernández-Hermógenes et al., 2017), or during use of non-interactive and interactive media (Suckfüll and Unz, 2016; Brill, 2019). However, for the given research question, only a limited, very basic T-pattern analysis was performed. Since the research focus was on instances in which a media event was either preceded or followed by a blink with greater-than-chance timing, only T-patterns with a length of two events, that is a level of 1, were searched for. This approach does not use the method’s full analytical capacity for structured data, but suits the current research purpose well. T-pattern analyses with identical search parameters were conducted in the Theme, (2017) 6 software for datasets with the participants’ individual, time-stamped blink data as user events, and either maxima in optical flow or coded scene-related events as media events. For each experimental group and each of the two types of media events, individual participant data files were imported into a Theme project, and were concatenated into a multi-sample data file, that is the series of all individual observation periods formed one virtual observation period. The level of significance for the critical interval was chosen as the standard *α* = 0.005, and only T-patterns were considered that appeared at least three times in a detection process. For validation purposes, the search process was repeated 40 times each with shuffled and rotated randomized versions of the data sets. Since the identical structure of media events during each observation leads to a large number of irrelevant patterns, only patterns including blinks were selected in detection results from real and randomized data.

## Results

### Manipulation Check

**Figure 2:**
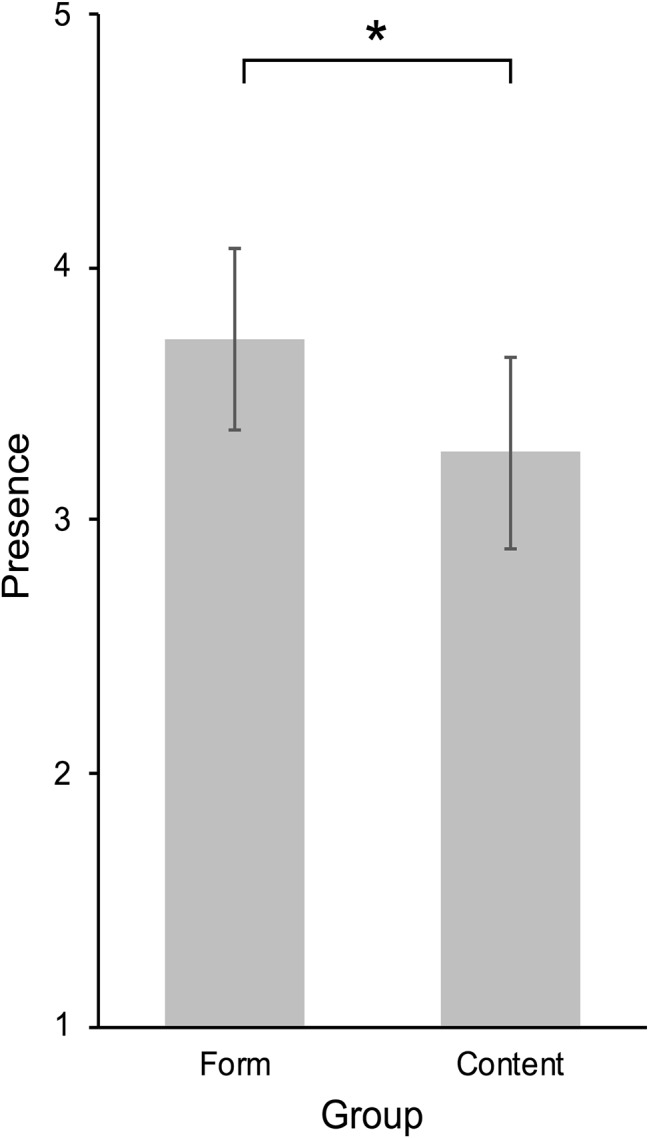
Spatial presence self-report scores in the form condition and content condition. Higher values indicate stronger presence experiences. Error bars indicate 95% CIs of the SEM. *p < 0.05.

We hypothesized that participants in the high presence condition, who were instructed to view the movie, report higher presence scores than participants in the low presence condition, who were explicitly instructed to focus on form aspects of the movie. The four item SPSL scale showed good internal consistency with a Cronbach’s alpha of *α* = 0.93. Since test requirements were fulfilled, a one-tailed independent samples *t*-test could be calculated and showed that, contrary to hypothesis, SPSL scores in the content group were not significantly higher than SPSL scores in the form group (*t*(47) = −1.69, *p* = 0.951, *d* = −0.48). In fact, participants in the form condition reported higher presence scores (content: *n* = 24, *M* = 3.27, SD = 0.95; form: *n* = 25, *M* = 3.72, SD = 0.92). A follow-up one-tailed independent samples *t*-test showed that this difference was statistically significant (*t*(47) = −1.69, *p* = 0.049, *d* = −0.48; see Figure 2).

Compliance with instructions was tested for both groups using the additional, corroborative manipulation check items. Since *t*-test requirements were not met, one-tailed Mann-Whitney U tests were calculated, and showed that participants in the form condition reported significantly higher attention to technical aspects than the content group (*U* = 153.50, *p* = 0.001, *d* = 0.30), but did not differ significantly from the content group on the other measures (attention to content aspects: *U* = 339.50, *p* = 0.198, *d* = 0.32; immersion into the mediated environment: *U* = 283.00, *p* = 0.647, *d* = 0.17; critical reception of the stimulus: *U* = 344.50, *p* = 0.827, *d* = 0.34).

### T-Pattern Analysis and Group Comparisons

#### Optical flow

**Figure 3:**
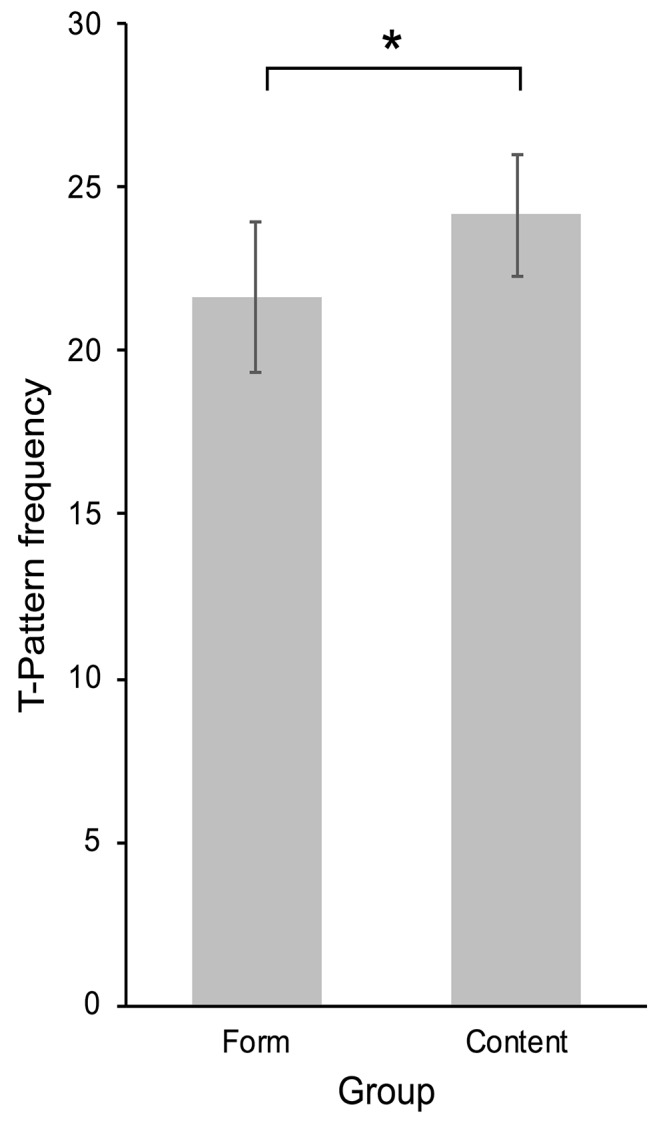
Mean frequencies of T-patterns with blinks and peaks in optical flow in the form condition and content condition. Error bars indicate 95% CIs of the SEM. *p < 0.05.

T-pattern analysis with consecutively numbered peaks in optical flow as media events, and blinks as user events identified 43 different T-patterns in the content group, with 580 combined occurrences, and 42 different T-patterns in the form group, with 541 occurrences. Comparison of detected T-patterns of length 2 in real data and randomized data showed that considerably more T-patterns were detected in real data (see Table 1), thus supporting the validity of the results. Individual pattern occurrences, that is the occurrences of all different patterns for each participant, were used for group comparisons of the high and low presence condition (high presence: *n* = 24, *M* = 24.17, SD = 5.72; low presence: *n* = 25, *M* = 21.64, SD = 4.76). The requirements for a one-tailed, independent samples *t*-test were met, and the test showed that significantly more T-patterns with blinks and media events were identified in participants in the content group (*t*(47) = 1.68, *p* = 0.050, *d* = 0.48; see Figure 3).

**Table 1:** T-patterns with blinks as viewer behavior and peaks in optical flow as media events in real data and randomized data.

Group	Real		Randomized				Difference in SD	
			Shuffle		Rotation			
	Unique	Total	*M*	SD	*M*	SD	Shuffle	Rotation
Content	43	580	13.06	3.53	17.43	3.76	8.47	6.80
Form	42	541	10.83	3.09	15.86	3.35	10.10	7.81

#### Cinematic structure

**Figure 4:**
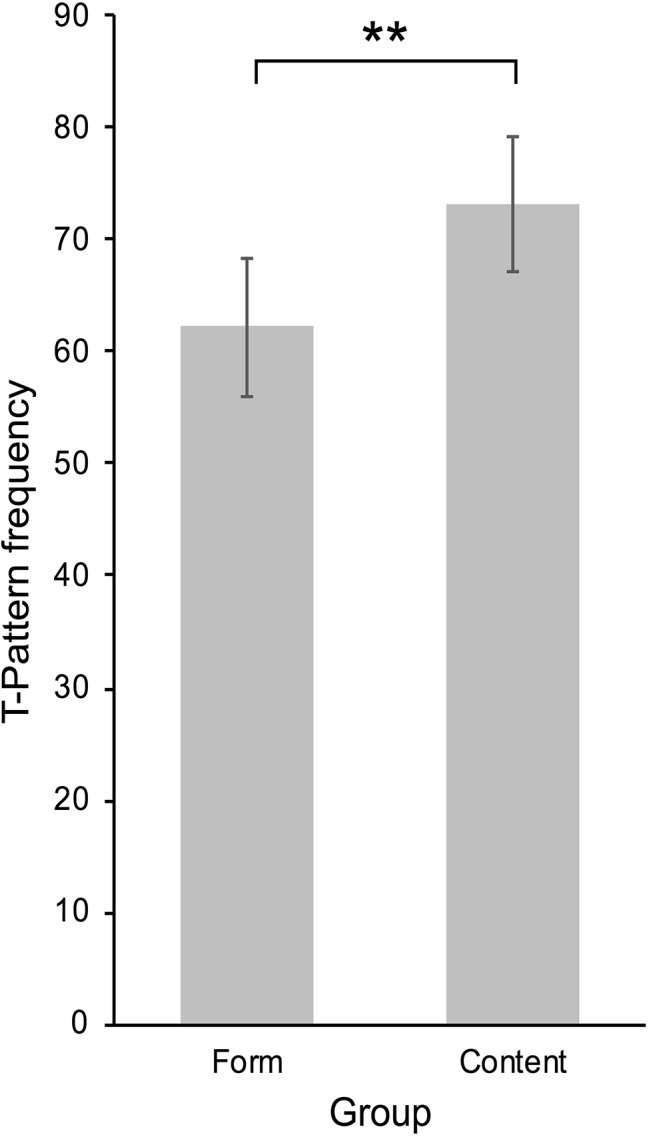
Mean frequencies of T-patterns with blinks and events of cinematic structure in the form condition and content condition. Error bars indicate 95% CIs of the SEM. **p < 0.01.

T-pattern analysis with consecutively numbered, scene-related changes as media events, and blinks as user events identified 139 different T-patterns with 1,758 combined occurrences in the content group, and 134 different T-patterns with 1,556 occurrences in the form group. Comparison of detected T-patterns of length 2 in real data and randomized data showed that considerably more T-patterns were detected in real data (see Table 2), thus supporting the validity of the results. Again, individual pattern occurrences for each participant were used for group comparisons of the high and low presence conditions (high presence: *n* = 24, *M* = 73.25, SD = 15.05; low presence: *N* = 25, *M* = 62.24, SD = 15.76). The requirements for a one-tailed, independent samples *t*-test were fulfilled. The test showed that significantly more T-patterns with blinks and media events were identified in participants in the content group [*t*(47) = 2.50, *p* = 0.008, *d* = 0.71; see Figure 4].

**Table 2:** T-patterns with blinks as viewer behavior and cinematic structure as media events in real data and randomized data.

Group	Real		Randomized				Difference in SD	
			Shuffle		Rotation			
	Unique	Total	*M*	SD	*M*	SD	Shuffle	Rotation
Content	139	1,758	43.23	49.60	59.80	65.72	15.03	13.39
Form	134	1,556	34.40	39.89	50.45	57.12	18.16	12.53

## Discussion

### Summary, Integration, and Meta-Inferences

The present study aimed at evaluating stimulus-dependent temporal structure in spontaneous eye-blink behavior as a possible alternative measure for spatial presence experiences. To this end, data from self-report, observation, and content analysis have been collected in an experiment with externally valid stimulus and setting, and two conditions with low and high presence potential. The various data have been integrated and analyzed to assess the degree of convergence between the objective indicator and an established self-report measure for presence. While analysis of data from observation and content analysis showed the predicted result of more stimulus-dependent structure in blinking behavior in the high presence condition, the different self-report measures showed divergent and unpredicted results. Participants in the low presence condition reported a stronger focus on the medium itself, but, at the same time, higher levels of presence experiences.

Thus, the mixed methods approach revealed an overall pattern of results that contradicts predictions of the underlying theory. According to the manipulation check items, participants in the low-presence condition paid more attention to stimulus form features, but according to the standardized SPES questionnaire, they also reported higher presence experiences. If all measures are valid, then this pattern of results is in conflict with the underlying presence theory, since presence should emerge from not paying attention to the medium itself, and should be diminished when viewers focus on the medium itself (e.g., Wirth et al., 2007; Liebold et al., 2017). Even when considering the high immersive potential of the experimental setup that may have interfered with the extended, media-focusing task in the low presence condition, the results are still not in line with the theory’s prediction. After all, the reported levels of presence were not equal to the natural viewing condition, but were significantly higher. On the other hand, T-pattern analysis of the observational data showed, as predicted, a higher degree of stimulus-dependent structure in blinking behavior in the high-presence condition. This result was found with two different approaches for the definition of media events. In addition, results that are not reported in this paper support the findings with another, media-event-independent analysis method for behavioral structure (Brill, 2019).

### Limitations and Future Directions

Aside from the study’s specific results, structured blinking behavior as a measurement approach for presence has limitations in itself due to its theoretical and conceptual foundations. First, structured blinking behavior is not directly influenced by presence experiences. It rather reflects the activity of attentional and other cognitive processes that should engage in processing of mediated information when recipients are in a state of presence, such as orienting responses on media content (Liebold et al., 2017). Like other suggested corroborative measures (e.g., Böcking et al., 2008), it could thus serve as an indirect indicator for presence by allowing to infer on presence-related processes. However, this limitation is inherent to all objective presence measurement approaches, since there is no known direct indicator for presence experiences. Even methodically sophisticated neuroimaging studies resort to observing indicators for presence-related processes, such as activation of brain areas for spatial processing and navigation, emotional processing, or executive control systems (Baumgartner et al., 2006). Second, attention is theorized to be not only a crucial component of presence formation, but also of other media use phenomena, so it would probably not be an indicator exclusively for presence. On the other hand, this may also broaden the applicability of a measure based on structure in blinking behavior. Third, attention is modeled as a necessary, but not sufficient condition for the emergence of presence (e.g., Wirth et al., 2007). However, at least in the present study, we found differences in behavioral structure in recipients who allocated their attention to the same stimulus, but with different intentions during reception. This suggests that the measure may in principle be sensitive to different attention on, and processing of a stimulus.

In the present study, two approaches have been used to identify relevant media events for T-pattern detection: optical flow as a basic, perception-related property of the stimulus, and media events related to the cinematic presentation of stimulus content. Future studies could extend the focus on media features further to the domain of media content by considering, for example, suspenseful media events and their relation to attentional focus, as has been addressed for the media use phenomenon of narrative transportation (Bezdek et al., 2015; Bezdek and Gerrig, 2017), or by considering media events with evolutionary and affective relevance (Brill et al., 2018). Regarding methodological aspects, T-pattern analysis offers the researcher several parameters to adapt the analysis to the specific behavior. The analytical approach in this study largely used the detection software’s standard analysis parameters that were, most importantly, identical for analysis of all experimental groups. However, the precision of analysis could possibly be improved further by adapting analysis parameters specifically to blinking behavior, for example regarding limits for the critical interval in T-pattern detection, or randomization procedures in validation of results.

### Conclusion

Different from a mono-method study design, the present study included data from several sources. Standardized self-report measures and computationally defined media events were included as purely quantitative measures. Theoretically derived media events were included as data that has been obtained by considering qualitative aspects during content analysis of the stimulus. Above all, our procedures of collecting observational data on viewer behavior, and the subsequent analysis of behavioral structure, can be regarded as an important component of a mixed methods approach (Anguera et al., 2018). Detailed reports on procedures of data collection, assessment of data quality, and data analysis were provided for both quantitative and qualitative measures (Venkatesh et al., 2013). Further, the employed mixed methods design is theoretically grounded (Venkatesh et al., 2013), since it has been derived from existing research on presence and on evaluations of alternative presence measurement approaches. An advantage of mixed methods research is the possibility to draw meta-inferences from the study results for the underlying theory (Venkatesh et al., 2013). In the present study, we aimed at a comprehensive description and explanation of recipient experience and behavior in a media use situation in order to evaluate a possible alternative measurement method. While this was not possible due to an unpredicted pattern of results, the study nevertheless provided new evidence on contradictions or boundary conditions of the underlying theory, an aspect that has been suggested as an essential outcome of mixed methods research (Venkatesh et al., 2013). In addition, the present study’s laboratory setup, stimulus, and measurement methods converge to a certain extent with human ethology’s calling for “direct, objective, non-intrusive and open-minded observation of each species’ behavior in its natural environment” (Magnusson, 2016). Further, the study follows an unconventional analysis approach by not only considering frequencies, rates, or sequences of behavioral units, but focusing on the temporal structure of behavior to describe a dynamic process phenomenon (Anguera et al., 2018).

While the overall, conflicting pattern of results does not allow for a conclusive answer to the original research question, the mixed methods approach nevertheless allowed for a level of insight into the research topic that could hardly be obtained in a mono-method study. Theory development on presence, and probably on other media use phenomena, as well, can benefit from more complex studies and measures that offer researchers a process perspective on the dynamic nature of media use phenomena.

## Data Availability

The datasets collected for this study are available upon request from the corresponding author.

## Ethics Statement

This study was carried out in accordance with the ethical guidelines of the German Psychological Society (Deutsche Gesellschaft für Psychologie, DGPs), the American Psychological Association (APA), and the Declaration of Helsinki. All participants provided written informed consent. The stimulus movie has been released for audiences as of 12 years of age by the German movie rating board (Freiwillige Selbstkontrolle der Filmwirtschaft, FSK), and was thus assessed as appropriate. Due to the use of standard stimulus material and measurement methods, no approval of the local ethics committee had been requested.

## Author Contributions

The major part of the study was conducted as part of the doctoral thesis of MB, with FS as thesis supervisor. MB and FS contributed conception and design of the study’s extension with content-analysis-defined media events, and MB contributed the additional analyses in this part. All authors contributed to manuscript drafting and revision, and have read and approved the submitted version.

### Conflict of Interest Statement

The authors declare that the research was conducted in the absence of any commercial or financial relationships that could be construed as a potential conflict of interest.
